# Evaluation of Post-Thaw Swim-Up Selection Combined With Glutathione Addition for Improvement of Sperm Chromatin Maturity in Normozoospermic Samples

**DOI:** 10.7759/cureus.40313

**Published:** 2023-06-12

**Authors:** Raghad Abd Albari Shimal, Amal Abdulwahid Mohammed, Essraa Mohsen Al-Essawe

**Affiliations:** 1 Department of Applied Embryology, High Institute for Infertility Diagnosis and Assisted Reproductive Technologies, Al-Nahrain University, Al-Nahrain, IRQ; 2 Department of Physiology, High Institute for Infertility Diagnosis and Assisted Reproductive Technologies, Al-Nahrain University, Al-Nahrain, IRQ

**Keywords:** dna fragmentation, normozoospermic men, asthenozoospermic, cryopreservation, glutathione

## Abstract

Background: Cryopreservation of human semen alters the spermatozoal structure, resulting in a reduction in sperm function parameters. Various antioxidants may be able to slow or prevent this type of injury. Glutathione (GSH) has numerous antioxidant properties; supplementing the semen with GSH before freezing may assist in the restoration of post-thaw sperm functionality.

Objective: To investigate the effect of adding 5mM of glutathione before freezing on human sperm cryosurvival.

Materials and methods: Semen samples were collected from 30 patients (22 normozoospermic and 8 asthenozoospermic) after 3-5 days of sexual abstinence. Following liquefaction, macro- and microscopic examinations were performed. The samples were then divided into two equal aliquots: the first aliquot received 5 mM of glutathione before freezing, and the second aliquot was considered the control (without glutathione). The samples were frozen using the rapid cryopreservation method and then preserved for 7-10 days in a liquid nitrogen tank before being thawed. Thawed samples from each group were examined microscopically according to the WHO 2010 guidelines. Aniline blue was used to assess the maturity of the DNA. Then, the direct-swim-up technique was applied to thawed samples from both groups to select the best sperm quality.

Results: The cryopreservation process negatively impacts all sperm characteristics in both groups. Sperm DNA integrity decreased. Glutathione addition before freezing decreased sperm chromatin immaturity (SCI) percent compared to the control (22.98 ± 0.83, 20.79 ± 0.56). The post-thaw performance of the swim-up technique resulted in a select sperm population with high progressive motility (p = 0.04) and an improvement in DNA integrity in the treated group versus the control group after thawing.

Conclusion: The DNA integrity in normozoospermic samples was improved by adding 5 mM glutathione before freezing. Performing the swim-up technique after thawing had no effect on sperm quality in asthenozoospermic patients.

## Introduction

Cryopreservation was discovered as a technique to maintain fertility in the 1960s. Currently, methods for keeping human spermatozoa frozen are widely applied in assisted reproduction [1]. In assisted reproductive technology (ART) clinics, the cryopreservation of human sperm is a common practice for a variety of reasons, including the preservation of fertility before cancer treatment [2]. Spermatozoa preservation to treat oligozoospermia and azoospermia, testicular sperm extraction (TESE) or percutaneous epididymal sperm aspiration (PESA) due to ejaculatory dysfunction or spinal cord damage, or sperm donation [3]. Sperm cryopreservation aims to preserve the structural integrity of the sperm before freezing while preserving its viability, motility, and fertilizing capacity [4].

Traditional freezing techniques do not need automated machinery. The sample is placed in liquid nitrogen at -196°C after coming into direct contact with nitrogen vapors at -80°C [5]. Major disadvantages of this method include its inability to manage the temperature decline curve. According to research by Tongdee and colleagues, quick freezing or slow programmable freezing of human sperm significantly reduced their DNA integrity, morphology, and motility [6].

Reactive oxygen species (ROS) production and physical-chemical damage caused by cryopreservation lower sperm viability and motility, which in turn decrease sperm reproductive potential. Antioxidants, when added to freezing fluid, can reduce the negative effects of ROS and save sperm from cryodamage [7]. Antioxidants are substances that, by scavenging generated free radicals, can prevent or decrease oxidative reactions [8]. In human semen cryopreservation, glutathione (GSH) is used as an antioxidant supplement for enhancing sperm motility by reducing ROS [9].

Glutathione is a thiol tripeptide (glutamyl cysteinyl glycine) that is found in the somatic cells and gametes of animals and has several biological functions. This thiol is essential for both endogenous and exogenous components to prevent oxidation [10,11]. Glutathione is the master detoxifier and antioxidant of the immune system [12,13]. The addition of 5 mM glutathione to human semen after thawing considerably increased sperm motility, vitality, and normal morphology in comparison to other concentrations used (2.5 and 10 mM) [14]. The goal of this study was to investigate the effect of adding 5 mM glutathione before freezing on human sperm cryosurvival in normozoospermic and asthenozoospermic semen samples. 

## Materials and methods

Thirty patients (normozoospermic and asthenozoospermic) between the ages of 23 and 44 provided sperm samples. The sample size was calculated based on the people visiting the place with a power of 0.8 and a 95 percent confidence interval. This study was self-funded due to budget and time constraints, as well as the fact that a sample size of 30 was determined to be statistically acceptable. Ethical clearance was obtained from Al Nahrain University with the number IEC/2020/222. After 3-5 days of sexual abstinence, the samples were acquired by masturbation in a clean, dry, and well-defined container. Semen samples were assessed macroscopically and microscopically in accordance with the WHO 2010 standard following liquefaction.

Blinding of the researcher who examined the samples was done so that there was no bias regarding the groups of individuals. There was convenient sampling in the study. Before being frozen, samples were divided into two equal pieces. The first sample was evaluated as a control with no additions (C group). 5 mM of glutathione was added to the second aliquot, which represented the treated group (T group).

Sperm samples were taken from individuals irrespective of the factors that govern their characteristics and frozen manually under controlled conditions using the Sperm FreezeTM Kit (Ferti Pro N.V., Industrie Park Noord 32, 8730 Beernem, Belgium). Sperm FreezeTM is a cryoprotectant composed of 26.76 percent glycerol in a HEPES buffer and 0.04 percent human serum albumin (HIV- and hepatitis-negative). The frozen substance required five minutes to reach room temperature. Subsequently, following the kit's instructions, 1 mL of the liquefied semen sample was placed in a cryovial, and 0.3 mL of the medium was supplied in a sperm freeze medium, dropwise, over about 5 minutes. The cryovials were then put in cryo-holders for 10 minutes to be cooled (-10°C). The samples were exposed to distant liquid nitrogen vapors at 20 cm (80°C) for 10 minutes before being immersed and stored in a liquid nitrogen tank [15]. After 7 to 10 days, the samples were taken from liquid nitrogen and put in cryovials for 10 minutes at ambient temperature, followed by 5 minutes in tap water until the ice melted.

Aniline blue staining (AB) is an acidic dye with a high affinity for non-lysine-rich histones that may be used to measure chromatin condensation [16]. AB was used to determine the DNA maturity of spermatozoa.

After thawing, the materials were centrifuged at a consistent speed and duration to execute the swim-up procedure (3000 rpm for 3 min). Before pouring 200L of new, pre-warmed flushing media onto the pellet, which was then incubated at 37 °C for 30-45 minutes, the supernatant was discarded.

The ethics committee of the High Institute for Infertility Diagnosis and Assisted Reproductive Technologies at Al-Nahrain University in Baghdad, Iraq, authorized the research. IBM Corp. Released 2015. IBM SPSS Statistics for Windows, Version 23.0. Armonk, NY: IBM Corp. was used to examine the data. The Kolmogorov-Smirnov test was used to check if the variables had a normal distribution. Before freezing, the mean values of normozoospermic and asthenozoospermic patients were compared using the independent samples t-test. The effect of 5 mM glutathione addition before freezing on sperm cryo-survival in normozoospermic and asthenozoospermic participants was compared using a two-way ANOVA followed by a Tukey honestly significant difference test. The variables were provided as the mean and standard deviation of the mean (mean SD). The significance threshold was fixed at p 0.05.

## Results

Based on each individual's sperm progressive motility (WHO 2010 guide), the samples were reclassified into two categories: asthenozoospermic (n = 9, 30%) and normozoospermic (n = 21, 70%); the sperm characteristics of both categories before cryopreservation are shown in Table 1. There were no statistical differences in age, abstinence days, liquefaction time, or semen volume. The normozoospermic group had higher sperm concentrations than the asthenozoospermic group (48.95 ± 4.75 vs. 34.00 ± 3.40 million/ml; P 0.02, respectively). The total motility and the progressive motility were higher in the normozoospermic group than in the asthenozoospermic group (P ≤ 0.001). Correspondingly, the percent of non-progressive motile spermatozoa and immotile spermatozoa was lower in the normozoospermic group versus the asthenozoospermic group (p ≤ 0.001). No significant differences in the percentage of normal sperm morphology and sperm chromatin immaturity (SCI) were shown in Table 1.

**Table 1:** Characteristics of samples involved in both groups NS: not significant. SE: standard error.

	Subjects included in this study			
Parameters	Normo-zoospermic Mean ± SE	Astheno-zoospermic Mean ± SE	p-value	Cut-off value
Age	31.67 ± 0.80	29.70 ± 1.35	0.20	
Days of abstinence	3.48 ± 0.16	3.56 ± 0.24	0.34	
Liquefaction time	33.67 ± 1.96	32.22 ± 1.69	0.56	30-60 minutes
Semen volume (mL)	2.80 ± 0.19	2.78 ± 0.28	0.75	≥ 1.5 mL
Sperm concentration	48.95 ± 4.75	34.00 ± 3.40	≤ 0.02	≥ 15x10^6^/mL
Total sperm count	133.45 ± 14.46	90.94 ± 10.11	≤ 0.02	≥ 39x10^6 ^/ ejaculate
Total motility (PR+NP)	79.05 ± 1.32	65.56 ± 1.94	≤ 0.001	40 %
Progressive motility (A+B)	49.61 ± 2.42	23.89 ± 2.32	≤ 0.001	≥ 30 %
Nonprogressive motility%	29.43 ± 2.13	41.67 ± 2.04	≤ 0.001	
Immotile spermatozoa%	20.95 ± 1.32	34.44 ± 1.94	≤ 0.001	
Total progressive motile spermatozoa%	79.79 ± 11.21	27.83 ± 6.90	≤ 0.001	
Normal sperm morphology %	37.48 ± 1.35	30.11 ± 3.43	0.25	
SCI%	20.79 ± 0.56	20.72 ± 1.69	0.16	

The effects of cryopreservation on sperm parameters according to normozoospermic and asthenozoospermic samples:

After thawing, total sperm motility percent and progressive motility percent decreased in both categories (p = 0.04). In normozoospermic men, the total motility decreased from 79.05% to 45.52%, while in asthenozoospermic men, it decreased significantly from 65.56% to 36.37%. (P ≤ 0.05) The normal morphology of sperm was also reduced in the normozoospermic group but not statistically in the asthenozoospermic. After thawing, SCI was higher in normozoospermic men than asthenozoospermic men, while there was no significant difference in SCI between groups in each category (p>0.05) Table 2.

**Table 2:** The effects of cryopreservation on sperm parameters of normozoospermic and asthenozoospermic samples NS: not significant. SE: standard error.

Variables	Treatment groups	Normozoospermic Mean ± SE	Asthenozoospermic Mean ± SE	P-value
Sperm concentration 10^6^/mL	Before	48.95 ± 4.75	34.00 ± 4.37	0.16
	After C	24.52 ± 2.59	20.22 ± 5.79	0.32
Total Motility %	Before	79.05 ± 1.32	65.56 ± 1.94	0.0001
	After C	45.52 ± 2.24	36.37 ± 3.62	0.04
Progressive %	Before	49.62 ± 2.42	23.89 ± 2.32	0.0001
	After C	21.72 ± 1.73	15.39±1.87	0.04
Non Progressive %	Before	29.43 ± 2.13	41.67 ± 2.04	0.002
	After C	23.80 ± 1.22	20.98 ± 2.34	0.78
Immotile sperm %	Before	20.95 ± 1.32	34.44 ± 1.94	0.0001
	After C	54.48 ± 2.24	63.63 ± 3.62	0.04
Normal Sperm Morphology %	Before	37.48 ± 1.35	30.11 ± 3.43	0.02
	After C	23.51 ± 2.36	18.63 ± 3.43	0.81
SCI %	Before	20.79 ± 0.56	20.72 ± 1.69	0.38
	After C	22.98 ± 0.83	18.67 ± 1.97	0.02

The effect of adding 5 mM glutathione before freezing on post-thaw sperm parameters in normozoospermic and asthenozoospermic samples:

When glutathione was added before freezing, there were no significant differences in the means of sperm concentration, total sperm motility, progressive motility, non-progressive motility, immotile sperm%, and normal sperm morphology% for both categories (normozoospermic and asthenozoospermic samples). The SCI% was lower in asthenozoospermic patient samples than in normozoospermic samples in the control group (p= 0.024). Adding 5 mM of glutathione before freezing reduced the SCI% in the normozospermic samples compared with the control group in the same category, while there was no difference in the asthenozoospermic samples (Table 3).

**Table 3:** The effect of adding 5mM of glutathione before freezing on post-thaw sperm quality of normozoospermic and asthenozoospermic samples SE: standard error

Variables	Treatment groups	Normo-zoospermic Mean ± SE	Astheno-zoospermic Mean ± SE	P-value
Sperm concentration 10^6^/mL	After C	24.52 ± 2.59	20.22 ± 5.79	0.22
	After T	21.76 ± 2.80	19.67 ± 5.03	0.34
Total Motility %	After C	45.52 ± 2.24	36.37 ± 3.62	0.036
	After T	47.19 ± 2. 45	44.11 ± 5.01	0.11
Progressive %	After C	21.72 ± 1.73	15.39 ± 1.87	0.04
	After T	21.29 ± 1.26	16.67 ± 1.59	0.04
Non Progressive %	After C	23.80 ± 1.22	20.98 ± 2.34	0.23
	After T	25.90 ± 1.89	27.44 ± 4.19	0.34
Immotile sperm %	After C	54.48 ± 2.24	63.63 ± 3.62	0.04
	After T	52.81 ± 2. 45	55.89 ± 5.01	0.44
Normal Sperm Morphology %	After C	23.51 ± 2.36	18.63 ± 3.43	0.56
	After T	24.00 ± 2.22	30.56 ± 3.26	0.23
SCI %	After C	22.98 ± 0.83	18.67 ± 1.97	0.02
	After T	19.26 ± 0.51	19.98 ± 1.45	0.22

There were no significant variations in the means of sperm concentration, total sperm motility, progressive motility, non-progressive motility, immotile sperm percentage, or normal sperm morphology percentage when glutathione was administered before freezing.

The SCI% was lower in asthenozoospermic patient samples than in normozoospermic samples in the control group (p= 0.024). Adding 5 mM of glutathione before freezing reduced the SCI% in the normozoospermic samples compared with the control group in the same category, while there was no difference in the asthenozoospermic samples.

## Discussion

The combined use of glutathione supplementation before cryopreservation and post-thaw swim-up selection has a positive impact on human sperm cryosurvival was the hypothesis tested. In the present investigation, samples were separated into two groups based on the proportion of raw sperm with increasing motility. Overall motility, progressive motility, non-progressive motility, and normal sperm morphology were all decreased in both groups after thawing (Table 2), which is similar to the results of [14]. Despite the fact that human spermatozoa are very resistant to cold shock, the freezing and thawing process causes considerable motility and viability [17]. Sperm chromatin immaturity (SCI%) was significantly lower in the asthenozoospermic group compared to the normozoospermic group; this may be due to variation in sperm motility at baseline onset, as higher motility in normozoospermic samples is associated with greater metabolism and higher ROS production [18,19]. The tiny sample size should also be taken into account. It was discovered that the fraction of spermatozoa with DNA fragmentation increased during storage and cryopreservation [20].

The addition of 5 mM glutathione before freezing had a noticeable effect on the motility metrics and normal morphology of sperm in either category. The progressive motility was significantly higher in normozoospermic samples than in asthenozoospermic samples. Glutathione levels in seminal plasma were found to influence fertility because glutathione protects the cellular membranes of the spermatozoa from oxidative damage [21]. The treated group had a considerably lower SCI percentage in the normozoospermic group than the control group. Estrada et al. (2017) [22] discovered that adding 2 mM GSH to the freezing solution of pig sperm increased spermatozoal nuclear stability and membrane integrity, enabling the sperm cells to tolerate freeze-thaw operations.

In research conducted by Rajimakers et al. [21], GSH levels in the seminal fluid of fertile males were shown to be considerably greater than those of sub-fertile men. On the other hand, it was observed that the addition of 5 mM GSH after freezing enhanced sperm motility characteristics, morphology, and viability, with the greatest improvement occurring in instances of asthenozoospermia [14]. While in vitro research has shown that GSH protects sperm qualities and parameters, there is insufficient data to recommend its usage in males with idiopathic male infertility [23].

During the freezing and thawing processes, Thomson et al. (2009) [24] found that too much ROS production and oxidative stress were important factors. During freezing, the formation of reactive oxygen species (ROS), which has been documented to cause alterations in membrane function and structure [25], and the modification of antioxidant defense mechanisms have both been reported to impact cell viability [26]. The effect of adding 5 mM glutathione before freezing on sperm parameters following the post-thaw swim-up selection technique of normozoospermic and asthenozoospermic samples.

In the present investigation, samples of sperm from males of similar ages with asthenozoospermia and normozoospermia were compared. The swim-up procedure was performed on thawed samples, and there were no significant variations between normo- and astheno-zoospermic sperm characteristics. Additionally, the current study revealed a significant and notable increase in the proportion of active motile sperm (progressive motility) and DNA integrity within both the normozoospermic and asthenozoospermic categories when comparing the control group to the treated group (Table 3). This outcome may be a consequence of glutathione's ability to promote the maturation of sperm chromatin. Glutathione is present both intracellularly (in sperm) and extracellularly (in seminal plasma) and functions as an essential endogenous non-enzymatic antioxidant. It is a sulfhydryl-containing tripeptide that plays a crucial role in oxidative damage and toxin defense. Its free sulphydryl groups may react directly with hydrogen peroxide, superoxide anion, and hydroxyl radicals and serve as a cofactor for glutathione peroxidase and glutathione S-transferase, enzymes that catalyze the reduction of harmful H2O2 and hydroperoxides [27].

Only active motile spermatozoa with normal morphology and excellent motility might be capable of swimming up to the upper layer [28,29]. The addition of GHS to the spermatozoa environment during cryopreservation as an antioxidant could be an effective strategy for preventing ROS-induced DNA damage and protecting the spermatozoa. Although this study found that there were no differences between asthenozoospermic and normozoospermic samples, which could be attributed to the small sample size, the effects of 5 mM GHS addition before freezing in whole samples and when the samples were divided into normozoospermic and asthenozoospermic categories followed the same pattern.

The study has some limitations that the researchers did not mention, such as using any corrections for multiple comparisons, such as the Bonferroni correction, which could help to control the family-wise error rate.

## Conclusions

In spermatozoa, ROS are generated metabolically and are necessary for sperm function. However, excess levels of ROS cause oxidative damage, such as membrane lipid peroxidation and DNA fragmentation, that adversely affects the early embryo and subsequent fetal development after fertilization. The current study concluded that the SCI% in normozoospermic samples was reduced by adding 5 mM glutathione before freezing. Adding glutathione before freezing and then performing the swim-up technique after thawing had no effect on sperm quality in asthenozoospermic patients.
